# How Does Active Learning Pedagogy Shape Learner Curiosity? A Multi-Site Mediator Study of Learner Engagement among 45,972 Children

**DOI:** 10.3390/jintelligence12060059

**Published:** 2024-06-05

**Authors:** Ji Liu, Dahman Tahri, Faying Qiang

**Affiliations:** Faculty of Education, Shaanxi Normal University, Xi’an 710062, China; dahmantahri@snnu.edu.cn (D.T.); fayingqiang@snnu.edu.cn (F.Q.)

**Keywords:** active learning pedagogy, learner engagement, learner curiosity, structural equation modeling

## Abstract

Curiosity is one of the most fundamental biological drives that stimulates individuals’ intense desire to explore, learn, and create. Yet, mechanisms of how curiosity is influenced by instructional pedagogy remain unclear. To shed light on this gap, the present study sets out to investigate the underlying channels linking active learning pedagogy, learner engagement, and learner curiosity, employing a partial least-squares structural equation model leveraging the Social and Emotional Skills Survey dataset across ten sites (N = 45,972). Findings indicate that active learning pedagogy is positively associated with learner engagement (std. β = 0.016, *p* = 0.005), but there lacks a significant direct effect on learner curiosity (std. β = −0.001, *p* = 0.738). Structural mediation results show that learner engagement is a key mediating channel linking active learning pedagogy and learner curiosity (std. β = 0.013, *p* = 0.005).

## 1. Introduction

Social and emotional skills refer to a range of abilities and skills that enable individuals to manage their emotions and behaviors and navigate diverse social environments (Grant 2017; Mahoney et al. 2021). Existing studies have shown that learners’ non-cognitive skills are as critical, if not more important than their cognitive abilities (Beck and Jackson 2022; Váradi 2022). In particular, curiosity is a key driver of student learning and can be defined as an emotional state and a cognitive desire to explore, inquire, and interact with the environment (Litman 2005; Schmitt and Lahroodi 2008), characterized by mobilizing a healthy emotional–motivational system that is related to awareness, search, and responsibility for individual development and successful interaction with the surrounding community (Kashdan et al. 2004). 

Due to different theories, measures, and scales implemented to assess curiosity, scholars have long struggled to establish a clear definitional consensus. As such, curiosity is commonly referred to as a motivation to seek out additional information to sufficiently fill the knowledge gap, particularly under conditions of uncertainty leading to increased explorational behaviors (Loewenstein 1994). Prior studies suggested that curiosity can be operationalized as both a state and a trait (Evans and Jirout 2023). In its state aspect, learners are motivated to seek information through exploration or asking questions that help them address their immediate knowledge gaps. In its trait aspect, learners’ curiosity is seen as a more enduring eagerness to learn, reflecting their consistent inclination toward curiosity across different situations (Jirout et al. 2022). Further research considered curiosity as a facet of openness that is characterized by receptiveness to new experiences. Within the context provided, individuals tend to have a strong desire for engagement in exploration and understanding (Von Stumm and Ackerman 2013).

From a developmental standpoint, curiosity is malleable, and it can be nurtured and honed, implying the important role of learning strategies (Donnellan et al. 2022; Guo et al. 2023; Tang et al. 2022; Walton et al. 2022). For this reason, a growing number of policy makers, education practitioners, and researchers have voiced that schools are the most promising venues for mobilizing students’ curiosity through social interactions between teachers and students, which has led to increasing interest in how learning strategies stimulate students’ non-cognitive skills, particularly in their curiosity (Jackson 2012).

Existing educational research often examines pedagogical practices from motivational, behavioral, and social perspectives (Nsenga 2022). In prior studies, active learning pedagogy is considered one effective way to promote learners’ curiosity. For example, a combined behavioral analysis and brain imaging studies have indicated that curiosity states were associated with activity in the striatum and inferior frontal cortex and that active learning pedagogy would promote individuals to exhibit high curiosity, resulting in increased activation of putamen and left inferior frontal cortex (Oudeyer et al. 2013). More important, recent evidence has emphasized that active learning pedagogy not only enhances curiosity but also contributes to the development of other important social and emotional skills. On the one hand, active learning pedagogy is considered to be a fundamental ingredient that can promote cognitive and emotional well-being, behavioral engagement, and social interactions for efficient education (Abry et al. 2017; Miller et al. 2011). One previous study showed that teacher instructional engagement in the classroom may significantly control students’ engagement in learning (Skinner and Belmont 1993). Hence, Wetzel and Farrow (2023) described teachers’ objectives in an active learning classroom as a channel to establish a learning environment in which knowledge is collaboratively constructed by both the teacher and students rather than solely delivered by the teacher. On the other hand, fostering learners’ sense of engagement through active learning pedagogy can significantly enhance learners’ curiosity, leading learners to deeper, meaningful learning. In conceptual terms, learner engagement is defined as enthusiasm, dedication, and focus on school activities that, in addition to social engagement, include engagement with cognitive, emotional, and behavioral aspects of the school environment (Wang and Hofkens 2020). According to previous studies, a sense of interest and curiosity comes from being cognitively, emotionally, behaviorally, as well as socially engaged (Shah et al. 2018). In this regard, there is growing evidence that engagement has a significant positive impact on curiosity, scholars have begun to recognize the importance of engagement in fostering curiosity and interest in learning (Shin and Kim 2019).

While the positive predictive link between active learning pedagogy and learner curiosity has been previously documented, few studies jointly evaluate the potential effect of active learning pedagogy on learner curiosity using multidimensional mediators such as learner engagement and examined the degree of their influence, nor have utilized large-scale data leveraging a multi-informant, multi-site, and multi-cohort research designs. 

In attempts to address these gaps in the literature, this study aims to examine the following:

(1) What is the relationship between active learning pedagogy and learner curiosity?
(2) To what extent does learner engagement mediate the link between active learning pedagogy and learner curiosity?

## 2. Literature Review

Active learning pedagogy is an instructional strategy that advocates for a shift from a teacher-centered to a student-centered approach, having a major influence on students’ attitudes and leading to increased engagement and improved learning outcomes (Chi and Wylie 2014; Han 2021; Inayat and Ali 2020; Prince 2004). Studies have shown a positive correlation between teachers’ practices and student development of social and emotional skills (Guo et al. 2023; Isohätälä et al. 2020) by activating learning processes and dynamics to create instructional environments that stimulate the search for knowledge and encourage engagement in the learning process (Freeman et al. 2014).

Teachers’ implementation of active learning pedagogy aims to actively engage students in learning, fostering their personal growth and enabling them to excel academically. A growing body of research has demonstrated that active learning pedagogy is conducive to the promotion of the development of social and emotional skills, specifically increases the level of curiosity and exploration of learners when they are actively involved in classroom activities (Clark et al. 2019; Mouromadhoni et al. 2019). Accordingly, in school contexts, researchers have typically placed greater emphasis on “engagement”, which is defined by educational researchers as a mental state in which students demonstrate all possible efforts willing to take on the responsibility of working on a task (Rotgans and Schmidt 2011), through which teachers provide students with a platform to actively participate, innovate, and construct their knowledge. Researchers and scholars have considered learner engagement as a multidimensional construct (Reeve et al. 2004; Schmidt et al. 2018) in which behavior, emotion, and cognition interact with and influence each other; a set of empirical studies has provided compelling evidence to support the relationships between the components of engagement. The interaction and reinforcement between these factors have a cumulative effect on total involvement (Li et al. 2010). 

Teacher’s implementation of active learning instructional strategies establishes an effective intervention to help learners develop their cognitive, social and emotional skills. It was found to be a more valuable to offer increased support and interventions to help students develop higher-order thinking skills. The malleability of cognitive skills can be observed when teachers shape their students’ outcomes through which they provide a platform to actively participate in the learning process by creating open-ended tasks, opportunities for self-expression, and facilitating group processes (Berry 2020; Pedler et al. 2020). Learners are inspired to explore subjects of interest when teachers use inquiry-based learning techniques in order to extend and enhance the learners’ ability to think critically and motivate them to ask questions, investigate, and explore topics of interest (Jensen and Lawson 2011). 

Similarly, active learning pedagogy has been found to play a crucial role in increasing learners’ social and emotional well-being. On the one hand, social interaction, similar to the way it is commonly described, refers to those skills related to social performance and collaboration, such as cooperation, sociability, and trust, that can play a significant role in the learners’ social engagement and fostering positive relationships with both teachers and peers (Keiler 2018), as well as in developing a sense of belonging within the school community (Guo et al. 2023). Students demonstrated heightened learning efficacy when they were active and engaged in meaningful, interactive, iterative, and joyful (Hirsh-Pasek 2022). Findings suggest that teachers can impact the dynamics of student relationships in classroom contexts by encouraging collaboration on school-related tasks, fostering compromise and negotiation skills, and encouraging sociability among students (Chernyshenko et al. 2018; Dufresne et al. 1996; Isohätälä et al. 2020). On the other hand, emotional engagement is defined as a range of responses within the classroom environment, such as their levels of interest, optimism, happiness, and anxiety (Skinner and Belmont 1993), and students’ attitudes toward their environment (e.g., school, teachers, and peers), is highly associated with a stable emotional supportive environment, in which teachers create sense of respect, support, and acceptance between them and their students, in addition to their relation with peers (Fredricks and Mccolskey 2012). Consequently, researchers found that students with higher degrees of emotional engagement, involvement, and enthusiasm about school are more likely to be optimistic and hopeful about their future and have better self-reported academic performance (Hodges 2020). Likewise, increasing students’ feelings of happiness and enthusiasm, and support was found to be strongly associated with their desire to explore, leading them to improve their academic achievements and performance outcomes (Salmela-Aro et al. 2017). 

Furthermore, active learning pedagogy encourages students to take increased responsibility in regulating their own behaviors by creating a supportive and engaging learning atmosphere, giving the students a chance where they select their choices when it comes to lesson plans, and establishing clear and consistent expectations for behaviors. That positive reinforcement, according to Young and Talanquer (2013), encourages their active participation in the classroom, creating a safe space where students feel comfortable expressing their thoughts, ideas, and questions without fear of judgment, engage in actions, and keep their commitment (Fredrickson 2001). 

More concretely, research has linked learner curiosity development to the employment of those strategies, placing learners at the center of learning, engaging them in learning, and motivating them to generate their own questions and find answers through research, problem-solving, and critical thinking (Tang et al. 2022); it also creates an empowering and supportive environment that is characterized by a strong teacher-student relationship based on trust, empathy, and understanding (Theobald et al. 2020). Similarly, the extent to which students perceive autonomy in their school environment has been linked to positive emotional outcomes (Hardre and Reeve 2003). To this end, the evidence above has shown that active learning pedagogy provides opportunities for students to engage in meaningful learning and classroom activities, where they are encouraged to ask questions, share their thoughts, and actively participate in class discussions (Bae et al. 2020; Bae and Lai 2020; Vargas et al. 2018). This, in turn, gives them a genuine desire to explore, learn, ask questions, investigate further, and independently explore related topics, as they are cognitively, emotionally, behaviorally, and socially engaged in creative activities (Dufresne et al. 1996; Fredricks et al. 2004). 

**Figure 1:**
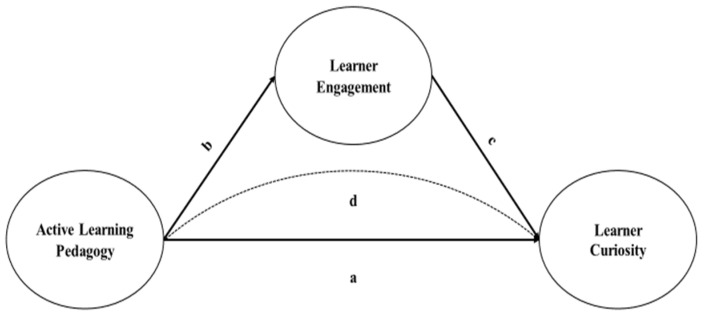
Conceptual model.

Given the critical multidimensionality of active learning pedagogy for learners’ curiosity and the lack of understanding about how learners’ engagement mediates the learning gains of adopting active learning pedagogy, the current study aims to understand how teachers’ implementation of active learning pedagogy shape learners’ curiosity (Figure 1, path “a”) and learner engagement (Figure 1, path “b”), as well as assess how learner engagement influences learner curiosity (Figure 1, path “c”). Also, it investigates the mediating role of learner engagement in the relationship between active learning and curiosity (Figure 1, path “d”), applying a partial least-squares structural equation model (PLS-SEM) using the SESS dataset.

## 3. Method

### 3.1. Sample and Participants

This study utilizes the publicly available Survey on Social and Emotional Skills (SSES), which was administered by the Organization for Economic Cooperation and Development (OECD) and released in 2021. The SSES is a large international investigation to identify and assess the conditions and practices that foster or hinder the development of social and emotional skills for age cohorts of ten- and fifteen-year-old students. To ensure sample representativeness, the first round of the SSES took place in 2018–2020 in ten cities across nine different countries, including Ottawa (Canada), Houston (USA), Bogota and Manizales (Columbia), Helsinki (Finland), Moscow (Russia), Istanbul (Turkey), Daegu (South Korea), Sintra (Portugal) and Suzhou (China), and the sample is designed to be globally representative of students in primary and secondary schools. The SSES questionnaire collects information on key socio-demographic indicators, which include gender, grade, academic achievement, and immigration background, while providing information on their parents’ level of education and socio-economic status. Besides, students’ parents, teachers, and principals also participated in the survey by providing all the necessary information on the students’ behaviors across the home and school environment (OECD 2021). 

**Figure 2:**
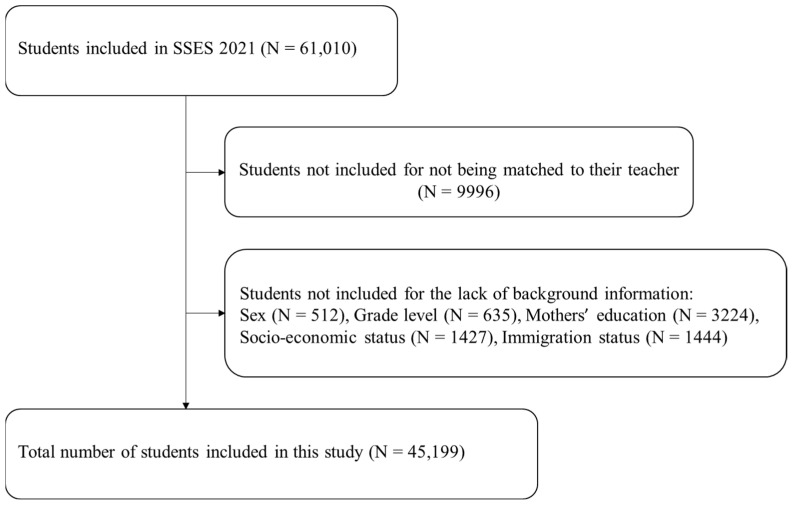
Flowchart of subject inclusion.

More specifically, inclusion criteria for study subject selection in this present study are defined as follows: (1) participated in the first wave of the SESS; (2) matched with the teacher or school students; (3) completed informed consent to participate. The resulting analytic sample involved 45,972 subjects who satisfied the inclusion criteria (Figure 2).

### 3.2. Measures and Instruments

#### 3.2.1. Active Learning Pedagogy

Active learning pedagogy is the key independent variable in this study. It is conceptualized as a latent construct that is measured utilizing a six-item scale adapted from the SSES teacher questionnaire. Items asked teachers how often these circumstances happen in their lessons: (1) students are given opportunities to explain their ideas; (2) a small group discussion between students takes place; (3) a whole class discussion takes place in which I participate; (4) I discuss questions that students ask; (5) students present something to the rest of the class; (6) students discuss materials from a textbook. Teachers’ responses were recorded on a four-point Likert scale, from which they could select: (1) “Never or almost never”, (2) “Some lessons”, (3) “Many lessons”, or (4) “Every lesson or almost every lesson”.

Particularly, construct validity indices and standardized factor loadings for active learning pedagogy are reported in Table 1. The six-item scale’s Cronbach’s alpha is recorded as 0.77, and the Kaiser–Meyer–Olkin (KMO) sampling adequacy test result is recorded as 0.90, both indicating good construct validity. Standardized factor loading results of the eight items are also presented in Table 1, which range from 0.58 to 0.77, indicating the reported results reflect that the latent construct is well supported.

#### 3.2.2. Learner Curiosity

Learner curiosity is the dependent variable in this study. For learner curiosity measurements, this study conceptualizes it as a latent variable and measure it relying on a six-item scale adapted from the SSES student questionnaire. The six-item questionnaire asked students whether they are curious about many different things, eager to learn, like to ask questions, like to know how things work, and love learning new things. Students’ responses were recorded on a five-point Likert scale, from which they could select: (1) “Strongly disagree”, (2) “Disagree”, (3) “Neither agree nor disagree”, (4) “Agree”, or (5) “Strongly agree”.

Construct validity indices for learner curiosity are reported in Table 2, where Cronbach’s alpha is recorded as 0.78, and the Kaiser–Meyer–Olkin (KMO) sampling adequacy test result is recorded as 0.83. These results show notable construct validity. Meanwhile, the scale’s standardized factor loading ranges from 0.57 to 0.80, indicating good latent construct support.

#### 3.2.3. Learner Engagement

Learner engagement is the mediator variable in this study. For measure validation, this study conceptualizes learner engagement as a latent construct and employed a twelve-item scale in the SESS student questionnaire for analysis. The scale includes four dimensions of “persistence”, “optimism”, “responsibility”, and “cooperation”, which are, respectively described in our study as (1) cognitive engagement; (2) emotional engagement; (3) behavioral engagement; and (4) social engagement, forming a total of twelve questions. Students’ responses were recorded and rated on a five-point Likert scale, from which they could select: (1) “Strongly disagree”, (2) “Disagree”, (3) “Neither agree nor disagree”, (4) “Agree”, or (5) “Strongly agree”.

Construct validity indices for learner engagement are reported in Table 3, where Cronbach’s alpha is recorded as 0.85, and the Kaiser–Meyer–Olkin (KMO) sampling adequacy test result is recorded as 0.91, both indicating an acceptable level of construct validity. The standardized factor loading results of the twelve-item are also presented in Table 3, which range from 0.54 to 0.68, indicating that the latent construct is supported.

#### 3.2.4. Correlation of Variables

To preliminarily assess the association among key variables in this study, a correlation analysis for all constructs is conducted. A positive correlation is observed between active learning pedagogy learner engagement (*ρ* = 0.412, *p* = 0.001) and learner curiosity (*ρ* = 0.226, *p* = 0.001). Similarly, results indicate a positive correlation between learner engagement and learner curiosity (*ρ* = 0.645, *p* = 0.001).

### 3.3. Data Analysis

In this study, all statistical analyses were performed using STATA version 15.1 (Stata, Stata Corp LLC, College Station, TX, USA) software. To achieve the research aims, a partial least-squares structural equation model (PLS-SEM) was built to examine the relationship between active learning pedagogy, learner curiosity, and learner engagement among 45,972 subjects in ten cities across nine different countries and fit a mediation model to evaluate the extent to which learner engagement act as a mediator in this relationship. From a methodological perspective, PLS-SEM offers several advantages over standard regression-based analytical approaches; one of the key methodological advantages is that researchers can precisely examine several sets of direct and indirect pathways, and the proposed hypotheses can be tested with empirical data, enabling its increasing application in the field of education. This present study utilizes PLS-SEM with the dual goal of minimizing the error term and maximizing explanatory power. In more specific terms, mediation tests are evaluated using three independent statistical tests, including Delta, Sobel, and Monte Carlo, with 5000 bootstrap samples. To achieve comparability, all variables are standardized to have a mean of zero and a standard deviation of one. 

## 4. Results 

### 4.1. Descriptive Analysis

Table 4 summarizes descriptive statistics of the 45,972 study subjects. Among them, the sexual composition of students is 51.0% female. In terms of grade level, students’ grades in this study ranged from grade 1 to grade 12, with an average grade-level at 7.00 (SD = 2.74). In terms of academic achievement, the arithmetic mean score on mathematics, reading, and art is calculated for each student, which shows a mean score of 57.08 (SD = 32.21) on a 100-point scale. In terms of parental education, mothers’ education levels at ISCED level 4 (upper-secondary school) or above is 57% of subjects. In terms of socio-economic status, a composite index of parental occupational status is computed with a global mean of 0.23 (SD = 0.99). Finally, 82% of subjects are native-born.

### 4.2. Mediation Analysis

Following the statistical analysis plan, goodness-of-fit statistics of the structural equation model is presented, with model fit measures CFI (Comparative Fit Index) = 0.941, TLI (Tucker–Lewis Index) = 0.922, RMSEA (Root Mean Square Error of Approximation) = 0.047, and SRMR (Standardized Root Mean Square Residual) = 0.041. CFI and TLI statistics indicating good model fit is 0.90, while RMSEA and SRMR statistics are below recommended upper-bound limits of 0.06 and 0.08, respectively. Therefore, it can be concluded that the model satisfies standard validity requirements (Hooper et al. 2008).

**Figure 3:**
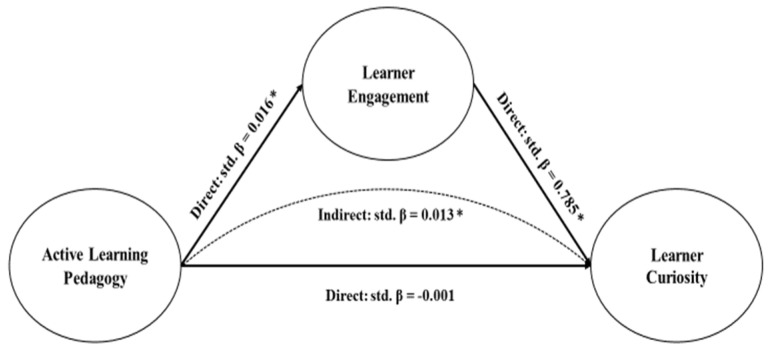
Standardized solution of the structural mediation model. * *p* < 0.05.

Second, this study evaluates both the direct and indirect effects of the structural mediation model utilizing results presented in Table 5 and Figure 3. For direct pathways, structural equation model findings show that active learning pedagogy is positively and significantly correlated with learner engagement (std. β = 0.016, *p* = 0.005), but there is no significant effect on learner curiosity (std. β = −0.001, *p* = 0.738). In addition, learner engagement is positively associated with learner curiosity (std. β = 0.785, *p* = 0.001). For indirect pathways, learner engagement significantly mediates the relationship between active learning pedagogy and learner curiosity (std. β = 0.013, *p* = 0.005). Considering that active learning pedagogy lacks a direct influence on learner curiosity, learner engagement fully mediates the relationship between active learning pedagogy and learner curiosity.

## 5. Discussion and Conclusions

This study sets out to assess the relationship between active learning pedagogy and learner curiosity and to evaluate the extent to which learner engagement plays a mediating role. While a set of empirical studies have supported the relationships between active learning pedagogy and socio-emotional academic outcomes (Han 2021; Prince 2004), examined the relationships between learner engagement and curiosity (Jirout and Klahr 2012), few studies have attempted to investigate the interconnected relationship between active learning pedagogy and socio-emotional outcomes via a mediation lens (Bonwell and Eison 1991; Shin and Kim 2019). Hence, the present research is designed to empirically examine the key mediating role of learner engagement. Findings from the structural mediation model show that while active learning pedagogy has a positive, non-significant direct effect on learner curiosity (std. β = −0.001, *p* = 0.738), active learning pedagogy is positively associated with learner engagement (std. β = 0.016, *p* = 0.005), and learner engagement is significantly corrected with learner curiosity (std. β = 0.785, *p* = 0.001). More important, results uncover an underexamined learner engagement channel through which active learning pedagogy can have a significant influence on learner curiosity (std. β = 0.013, *p* = 0.005).

One novel finding in this study is that active learning pedagogy is beneficial for enhancing learner engagement. Importantly, active learning pedagogy was found to foster the four dimensions of learner engagement: cognitive, emotional, behavioral, and social by encouraging learners to take increased responsibility inside or outside the classroom (Ben Izhak et al. 2023; OECD 2018; Pietarinen et al. 2014). To this end, engaged learners demonstrated higher degrees of cognition, positive emotions, interaction, and responsibility towards their environment, including active participation alongside teachers in effective learning strategies (Li and Lerner 2013). Notwithstanding, a key limitation of the present study is the inability to draw causal conclusions about the influence of pedagogy on curiosity. While novel findings in this study provide insights into a deeper understanding of the links between teaching pedagogies and cognitive and non-cognitive skills, future research utilizing instructional observation methods and objective measures is needed to fully understand the degree to which active learning pedagogy influences social and emotional outcomes.

Consistent with previous research, this study shows that the multi-dimensionality of learner engagement, including cognitive, emotional, behavioral, and social, is closely related to fostering learner curiosity (Hardre and Reeve 2003). These findings are in support of previous research, such as Guo et al. (2023), which highlighted a significant connection between learner cognitive engagement and the elicitation of learner curiosity. Furthermore, a supportive learning atmosphere of emotionally engaged learners in the classroom, associated with positive emotions, significantly promotes their curiosity in exploring and discovering new knowledge (Noordewier and Van Dijk 2017). In lieu of the above evidence, teachers can create an instructional environment conducive to fostering curiosity and a thirst for knowledge by incorporating specific pedagogical strategies (Di Leo et al. 2019; Grossnickle 2016; Jirout and Klahr 2012). Consequently, teachers who make a conscious effort to encourage learner questioning and exploration, provide opportunities for student-led investigations, and value students’ intrinsic motivations and interests are likely to have a significant influence on learners’ curiosity development in the long run.

## 6. Limitations and Potentials for Future Research

Finally, results of the present study cannot be fully interpreted without considering its limitations. The mediation findings generated from cross-sectional design would probably limit the establishment of causation and require careful interpretation. Accordingly, future researchers may use longitudinal or cross-lagged design studies to better understand the link between active learning pedagogy, learner engagement, and learner curiosity. Relatedly, another likely limitation should be acknowledged that active learning pedagogy in this study relied on self-reports of teachers without observational data to triangulate their support. For future research, it is recommended to incorporate direct observations to improve the validity of the variables. Finally, there may be some concerns about the nesting of teacher-to-student data, which is common to most educational settings, and that future studies may wish to more accurately account for in statistical analyses.

## Figures and Tables

**Table 1:** Latent measurement characteristics for Active Learning Pedagogy.

Items		Mean (SD)	Factor Loading
Active Learning Pedagogy (ALP)			
	Students are given opportunities to explain their ideas (TCQM01301)	3.43 (0.67)	0.64
	A small group discussion between students takes place (TCQM01302)	2.93 (0.79)	0.77
	A whole class discussion takes place in which I participate (TCQM01303)	2.91 (0.85)	0.76
	I discuss questions that students ask (TCQM01304)	3.15 (0.78)	0.72
	Students present something to the rest of the class (TCQM01305)	2.66 (0.80)	0.63
	Students discuss materials from a textbook (TCQM01306)	2.66 (0.93)	0.58

Note: Standard deviations are in parentheses. Cronbach alpha is 0.77, Kaiser–Meyer–Olkin measure of sampling adequacy is 0.90, and Bartlett’s test-of-sphericity statistic is 74,912.39 (df = 15, *p* = 0.001).

**Table 2:** Latent measurement characteristics for Learner Curiosity.

Items		Mean (SD)	Factor Loading
Learner Curiosity (LC)			
	I am curious about many different things (STA0401)	4.12 (0.90)	0.57
	I am eager to learn (STA0402)	3.92 (0.96)	0.74
	I like to ask questions (STA0403)	3.67 (1.06)	0.62
	I like to know how things work (STA0404)	4.08 (0.86)	0.67
	I like learning new things (STA0405)	4.23 (0.84)	0.80
	I love learning new things in school (STA0407)	3.95 (0.96)	0.76

Note: Standard deviations are in parentheses. Cronbach alpha is 0.78, Kaiser–Meyer–Olkin measure of sampling adequacy is 0.83, and Bartlett’s test-of-sphericity statistic is 91,826.08 (df = 15, *p* = 0.001).

**Table 3:** Latent measurement characteristics for Learner Engagement.

Items		Mean (SD)	Factor Loading
Cognitive Engagement (COE)			
	Keep working on a task until it is finished (STA1301)	3.83 (0.98)	0.63
	Finish what I start (STA1304)	3.82 (0.95)	0.67
	Finish things despite difficulties on the way (STA1310)	3.75 (0.94)	0.66
Emotional Engagement (EME)			
	Wake up happy almost every day (STA0903)	3.34 (1.21)	0.58
	Always positive about the future (STA0904)	3.76 (1.08)	0.59
	Look at the bright side of life (STA0906)	3.81 (1.05)	0.65
Behavioral Engagement (BEE)			
	Reliable and can always be counted on (STA0702)	4.02 (0.91)	0.54
	Keep my promises (STA0705)	4.14 (0.80)	0.60
	A responsible person (STA0706)	3.87 (0.92)	0.68
Social Engagement (SOE)			
	Get along well with others (STA1803)	4.03 (0.86)	0.60
	Always willing to help my classmates (STA1807)	3.99 (0.91)	0.63
	Polite, courteous to others (STA1809)	4.05 (0.85)	0.61

Note: Standard deviations are in parentheses. Cronbach alpha is 0.85, Kaiser–Meyer–Olkin measure of sampling adequacy is 0.91, and Bartlett’s test-of-sphericity statistic is 14,130.431 (df = 66, *p* = 0.001).

**Table 4:** Demographic characteristics of 45,972 study subjects.

Background Variables	Definition	M	SD
Female	Female = 1, Male = 0	0.51	-
Grade Level	Student grade level (1–12)	7.00	2.74
Academic Achievement	Arithmetic mean of student test scores on math, reading, and arts (0–100)	57.08	32.21
Mother’s Education	ISCED 4 or above = 1, ISCED 3 or below = 0	0.57	-
Socio-Economic Status	Composite index of parental occupational status (0–1)	0.23	0.99
Native	Native = 1, immigrant = 0	0.82	-

**Table 5:** Structural equation model results.

Effect	Std. β	Z	P	[95% Conf. Interval]
Direct effects				
ALP → LE	0.016	2.810	0.005	[0.005, 0.027]
ALP → LC	−0.001	−0.330	0.738	[−0.010, 0.007]
LE → LC	0.785	268.950	0.001	[0.780, 0.791]
Indirect effect				
ALP → LE → LC	0.013	2.813	0.005	[0.004, 0.021]

Note: Model fit measures are CFI = 0.941, TLI = 0.922, RMSEA = 0.047, and SRMR = 0.041.

## Data Availability

Data is publicly available at https://www.oecd.org/education/ceri/social-emotional-skills-study/ (accessed on 1 June 2023).
